# Exploration of Multi-Component Vanadium and Titanium Pnictides Using Flux Growth and Conventional High-Temperature Methods

**DOI:** 10.3389/fchem.2019.00909

**Published:** 2020-01-10

**Authors:** Alexander Ovchinnikov, Svilen Bobev

**Affiliations:** ^1^Department of Chemistry and Biochemistry, University of Delaware, Newark, DE, United States; ^2^Department of Materials and Environmental Chemistry, Stockholm University, Stockholm, Sweden

**Keywords:** flux growth, pnictides, crystal structure, electronic structure, metal clusters

## Abstract

The flux growth method was successfully employed to synthesize millimeter-sized single crystals of the ternary barium vanadium pnictides Ba_5_V_12_As_19+x_ (*x* ≈ 0.02) and Ba_5_V_12_Sb_19+x_ (*x* ≈ 0.36), using molten Pb and Sb, respectively. Both compositions crystallize in space group *P*$\bar{4}$3*m* and adopt a structure similar to those of the barium titanium pnictides Ba_5_Ti_12_*Pn*_19+x_ (*Pn* = Sb, Bi), yet with a subtly different disorder, involving the pnictogen and barium atoms. Attempts to obtain an arsenide analog of Ba_5_Ti_12_*Pn*_19+x_ using a Pb flux technique yielded binary arsenides. High-temperature treatment of the elements Ba, Ti, and As in Nb or Ta tubes resulted in side reactions with the crucible materials and produced two isostructural compositions Ba_8_Ti_13−x_*M*_*x*_As_21_ (*M* = Nb, Ta; *x* ≈ 4), representing a new structure type. The latter structure displays *fcc*-type metal clusters comprised of statistically distributed Ti and *M* atoms (*M* = Nb, Ta) with multi-center and two-center bonding within the clusters, as suggested by our first-principle calculations.

## Introduction

The application of molten metals as inert or reactive solvents for the crystal growth of intermetallic compounds is an actively used synthetic approach in solid-state chemistry. In a typical flux growth experiment, a mixture of selected metallic elements, along with a suitable flux material (also a metal or eutectic mixture of metals) are loaded in an inert container (graphite, alumina or other refractory materials, or high-melting metals from groups 5 or 6, such as Nb, Ta, and Mo). The reaction container can be equipped with some sort of filter or sieve for separating the molten flux from the grown crystals (Canfield and Fisk, 1992; Kanatzidis et al., 2005; Latturner, 2018). The as-prepared mixtures are then heated up, typically well above the melting point of the flux to achieve reasonably high diffusion rates and solubility. In general, complete dissolution is not necessary for production of small crystals, suitable for, e.g., single-crystal X-ray diffraction. However, obtaining larger crystals, which can be utilized for accurate physical property measurements, usually requires precipitation from homogeneous melts to assure a small number of crystallization centers (Wolff and Mlavsky, 1974). After the homogenization step, the temperature is lowered to allow formation of a solid phase. The flux material can then be removed by inverting the reactor at a temperature above the flux melting point and letting the molten flux pass through the filter, leaving the grown crystals behind. Alternatively, selective etching or evaporation of the flux can be used to recover the crystals from the reaction batch. In certain cases, single crystals can be mechanically isolated after breaking the solidified product (Canfield and Fisk, 1992; Kanatzidis et al., 2005). An important point that has to be taken into account when growing single crystals via the flux growth method is possible undesirable incorporation of the flux material into the crystal structure of the produced crystals. In such instances, the observed crystal structure may be inherently flux-stabilized and cannot be reproduced in a flux-free environment (Khan et al., 2018; Felder et al., 2019).

The flux growth process is well-suited for the production of incongruently-melting materials, but it can also be efficient for the growth of congruently-melting compounds when their melting points are prohibitory high. Since ternary and higher multicomponent phase diagrams are usually unknown, making the information about melting temperatures as well as phase relationships inaccessible, the flux method, owing to its wide applicability, offers a tool for explorative search for new materials. Various classes of intermetallics have been prepared by this route. In particular, numerous ternary (Ovchinnikov et al., 2017b; Baranets et al., 2019a; Childs et al., 2019; Nakamura et al., 2019; Ovchinnikov and Bobev, 2019a), quaternary (Ovchinnikov et al., 2017a, 2018a; Baranets and Bobev, 2019; Baranets et al., 2019b; Jayasinghe et al., 2019; Wakiya et al., 2019), and even quinary (Zaikina et al., 2010, 2017; Ma et al., 2012) compositions were successfully synthesized in the form of single crystals. Some examples of complex intermetallics which have been recently produced from metal fluxes include *AE*_3_Ti_8_Bi_10_ (Ovchinnikov and Bobev, 2019b), *AE*Li_2_In_2_Ge_2_ (*AE* = Sr, Ba, Eu) (Ovchinnikov and Bobev, 2019c), Y_7_Ru_4_InGe_12_ (Bao et al., 2019), and *R*_5_Mg_5_Fe_4_Al_*x*_Si_18−x_ (*R* = Gd, Dy, Y) (Ma et al., 2012).

Even apparently well-established binary systems may sometimes yield surprising new compositions, as it was the case for the *RE*–Bi (*RE* = Nd, Sm) systems (Ovchinnikov et al., 2018b), which host the isostructural *RE*_3_Bi_7_ phases, overlooked in the multiple early assessments of the corresponding phase diagrams. Careful optimization of the flux growth procedure resulted in large single crystals of these recently discovered compounds.

In this contribution, we explored the application of the metal flux approach to the synthesis of multinary vanadium pnictides. The choice of the elements was in part motivated by the lack of detailed experimental data on the ternary compounds in the *A*–V–*Pn* systems, where *A* stands for an electropositive metal, such as alkali, alkaline-earth, or rare-earth metal, and *Pn* is pnictogen, i.e., a group 15 element. From the physical property perspective, some of the early transition metal pnictides were reported to display interesting electronic behavior, such as superconductivity in K_2_Cr_3_As_3_ (Bao et al., 2015) and doped BaTi_2_Sb_2_O (Doan et al., 2012), and itinerant ferromagnetism in LaCrSb_3_ (Raju et al., 1998). In addition, our recent exploratory work in the field of titanium pnictides revealed several new phases in the *AE*–Ti–Bi systems (*AE* = Ca, Sr, Ba, Eu) (Ovchinnikov and Bobev, 2018a, 2019b). Taking into account pronounced structural similarities between multinary titanium and vanadium pnictides (Brylak and Jeitschko, 1994; Bie and Mar, 2009; Failamani et al., 2015; Ovchinnikov and Bobev, 2018a), the existence of yet unknown multinary vanadium pnictides can be foreseen.

We focused our studies on the ternary arsenides and antimonides of vanadium with alkaline-earth metals. Due to extremely low solubility of V in liquid Bi and the lack of stable binary bismuthides, Bi was excluded from our consideration, although it is conceivable that with suitably modified synthetic protocols, analogous bismuthides could also be found. The exploratory search resulted in two ternaries, Ba_5_V_12_As_19+x_ (*x* ≈ 0.02) and Ba_5_V_12_Sb_19+x_ (*x* ≈ 0.36), grown from liquid Pb and Sb, respectively. By high-temperature treatment of the elemental mixture with the nominal composition “Ba_5_Ti_12_As_19_” in Nb or Ta tubes, new isostructural compounds with the general formula Ba_8_Ti_13−x_*M*_*x*_As_21_ (*M* = Nb, Ta) were produced. A striking structural peculiarity of these phases, adopting a new structure type, is the presence of *fcc*-type transition metal clusters with pronounced multi-center, as well as two-center, bonding.

## Experimental

### Synthesis

Due to the air-sensitivity of the starting materials and the final products, most operations were performed in an argon-filled glovebox. All used materials had stated purity of at least 99.9 wt. %. Flux-assisted single crystal growth was employed for the synthesis of Ba_5_V_12_As_19.02(1)_ and Ba_5_V_12_Sb_19.36(2)_. For the former compound, metallic Pb was utilized as a flux. A mixture of Ba, V, As, and Pb with a molar ratio of 2:1:4:25, respectively, was placed in an alumina crucible topped with a piece of quartz wool and sealed in an evacuated fused silica tube. The reactor was heated up to 1173 K in a box furnace with a rate of 200 K/h. After the mixture was allowed to homogenize for 48 h, it was cooled down to 823 K with a rate of 5 K/h. At this final temperature, the fused silica tube was removed from the furnace, inverted, and subjected to centrifugation. The molten lead seeped through the quartz wool leaving the crystallized product on the wool and inside the crucible. The tube was break-opened inside the glovebox and the solid material was mechanically collected and inspected under an optical microscope. Besides some inhomogeneously looking powder, which was revealed by powder X-ray diffraction to be a mixture of binary Ba and V arsenides, well-formed single crystals of isotropic polyhedral shape and dimensions up to 1 mm were clearly visible. These crystals turned out to be a new compound with the composition Ba_5_V_12_As_19.02(1)_ as was determined by single-crystal X-ray diffraction.

The single crystal growth of Ba_5_V_12_Sb_19.36(2)_ was carried out applying a Sb self-flux approach. A similar experimental setup was used as detailed above. The starting mixture consisted of Ba, V, and Sb in a molar ratio of 2:1:4, respectively. The reactor was heated up to 1273 K with a rate of 50 K/h and kept at this temperature for 48 h. After that, the temperature was lowered to 1023 K with a rate of 5 K/h, and the excess of Sb was removed by centrifugation. Large crystals with dimensions up to 4–5 mm were the only solid product found in the crucible. Powder and single crystal X-ray diffraction analysis showed that the product was single phase Ba_5_V_12_Sb_19.36(2)_.

Crystals of the new quaternary compound Ba_8_Ti_9.24(6)_Ta_3.76_As_21_ were discovered in the sample prepared by a direct reaction of the elements with the targeted composition Ba_5_Ti_12_As_19_. The starting mixture was placed in a weld-shut Ta tube under protective high-purity Ar atmosphere and sealed in an evacuated fused silica tube, which was subsequently loaded in a tube furnace. One end of the tube was left protruding out from the furnace to keep this part of the reactor at room temperature, as a safety measure: in the case of the Ta tube failure, the toxic As vapor would condense on the cold end without building dangerously high pressure inside the silica tube. The tube was heated up to 1273 K with a rate of 200 K/h, annealed at this temperature for 48 h, and cooled down to room temperature with a rate of 5 K/h. The resulting sample mostly consisted of binary Ba and Ti arsenides according to powder X-ray diffraction. However, strong peaks belonging to an unidentified phase were evident in the powder patterns. Visual inspection of the product under a microscope revealed the presence of single crystals with different morphologies. Among them, small submillimeter-sized crystals with cubic or octahedral shape could be easily distinguished. Subsequent single crystal data collection and refinement confirmed a new structure with the chemical formula Ba_8_Ti_9.24(6)_Ta_3.76_As_21_. The Ta in the composition obviously originated from the reactor. To indirectly cross check the results of the structural refinement, the reaction was repeated using the same protocol as described above, but in a Nb tube instead of Ta. The outcome of the synthesis with respect to the major phases was similar to the previous attempt. Single crystals with the refined composition Ba_8_Ti_9.0(3)_Nb_4.0_As_21_, isotypic with the Ta-containing phase, were found as a side product, thus confirming the crystal structure.

### Powder X-Ray Diffraction (PXRD)

Powder X-ray diffraction measurements were done in the reflection mode on a Rigaku Miniflex diffractometer (Cu K_α_ radiation λ = 1.5418 Å) operating inside a nitrogen-filled glovebox to prevent sample deterioration upon contact with the ambient atmosphere. The powder patterns were recorded in a *Θ*–*Θ* scan mode with a step size of 0.05° and a 2 s per step acquisition time.

### Single-Crystal X-Ray Diffraction

Suitable single crystals were selected and cut to desired dimensions under dry Paratone N oil using low-background plastic loops. Data were recorded in a cold nitrogen stream at *T* = 200 K on a Bruker SMART APEX CCD diffractometer equipped with monochromated Mo K_α_ radiation (λ = 0.71073 Å). The raw data were integrated using the program SAINT (SAINT, 2014). Semiempirical absorption corrections were performed with the SADABS software (SADABS, 2014). Crystal structures were solved by dual-space methods with SHELXT (Sheldrick, 2015a) and refined by full matrix least-squares methods on *F*^2^ using SHELXL (Sheldrick, 2015b). All studied compounds showed a certain degree of crystallographic disorder. Assignment of chemical types was straightforward, based on the interatomic distances and coordination environments. Atomic coordinates were standardized using STRUCTURE TIDY (Gelato and Parthé, 1987). Details of the data collection, crystallographic parameters, and selected interatomic distances are summarized in Tables 1–6.

**Table 1:** Data collection details and selected crystallographic data (*T* = 200 K, Mo K_α_, λ = 0.71073 Å).

**Refined composition**	**Ba_**5**_V_**12**_As_**19.02(1)**_**	**Ba_**5**_V_**12**_Sb_**19.36(2)**_**	**Ba_**8**_Ti_**9.0(3)**_Nb_**4.0**_As_**21**_**	**Ba_**8**_Ti_**9.24(6)**_Ta_**3.76**_As_**21**_**
fw/ g mol^−1^	2722.96	3655.06	3474.22	3795.01
Space group	*P*$\bar{4}$3*m*	*P*$\bar{4}$3*m*	*Fm*$\bar{3}$*m*	*Fm*$\bar{3}$*m*
*Z*	2	2	4	4
*a*/ Å	11.398 (1)	12.125 (1)	15.684 (7)	15.691 (3)
*V*/ Å^3^	1480.8 (5)	1782.7 (5)	3858 (5)	3863 (2)
ρ_calc_/ g cm^−3^	6.11	6.81	5.98	6.52
μ_MoKα_/ cm^−1^	312.0	227.5	287.7	382.3
R_1_ [*I* > 2σ(*I*)]^a^	0.025	0.021	0.036	0.025
*w*R_2_ [*I* > 2σ(*I*)]^a^	0.054	0.043	0.080	0.065
R_1_ [all data]^a^	0.027	0.022	0.050	0.028
*w*R_2_ [all data]^a^	0.055	0.043	0.083	0.066
Flack's parameter	0.06 (2)	−0.03 (7)	—	—
Δρ_max, min_/ e Å^−3^	0.69−1.41	0.80−0.91	1.29−1.07	1.85−1.38

a *R_1_ = ∑||F_o_|–|F_c_||/∑|F_o_|; wR_2_ = [∑[w($F_{\text{o}}^{2}$-$F_{\text{c}}^{2}$)^2^]/∑[w($F_{\text{o}}^{2}$)^2^]]^1/2^, where w = 1/[σ^2^$F_{\text{o}}^{2}+$(AP)^2^+(BP)], and P = ($F_{\text{o}}^{2}+$2$F_{\text{c}}^{2}$)/3; A, B are the respective weight coefficients as in the corresponding CIFs (depository numbers 1959265-1959268)*.

**Table 2:** Atomic coordinates and equivalent isotropic displacement parameters for Ba_5_V_12_As_19.02(1)_.

**Atom**	**Site**	***x***	***y***	***z***	**$U_{\text{eq}}^{\text{a}}$ (Å^**2**^)**
Ba1A^b^	6*g*	0.1754 (3)	1/2	1/2	0.0109 (4)
Ba1B^b^	6*g*	0.208 (2)	1/2	1/2	0.0109 (4)
Ba2	4*e*	0.83313 (7)	*x*	*x*	0.0108 (3)
V1	12*i*	0.1576 (1)	*x*	0.3240 (2)	0.0081 (4)
V2	12*h*	0.1910 (2)	1/2	0	0.0073 (4)
As1	12*i*	0.21596 (8)	*x*	0.5341 (1)	0.0100 (3)
As2	12*i*	0.32944 (8)	*x*	0.0124 (1)	0.0079 (3)
As3	6*f*	0.3724 (2)	0	0	0.0076 (4)
As4^b^	4*e*	0.1123 (1)	*x*	*x*	0.0085 (6)
As5	4*e*	0.3145 (1)	*x*	*x*	0.0105 (5)
As6^b^	1*b*	1/2	1/2	1/2	0.010^c^
As7^b^	1*a*	0	0	0	0.010^c^

^a^*U_eq_ is defined as one third of the trace of the orthogonalized U_ij_ tensor*.

b *Refined occupancies, occ(Ba1A) = 0.90(1), occ(Ba1B) = 1 – occ(Ba1A), occ(As4) = 1 – occ(As7), occ(As6) = 1 – occ(Ba1A), occ(As7) = 0.020(7)*.

c *For As6 and As7, displacement parameters were refined isotropically and were constrained to the equivalent value U_eq_ of As5^1^*.

**Table 3:** Atomic coordinates and equivalent isotropic displacement parameters for Ba_5_V_12_Sb_19.36(2)_.

**Atom**	**Site**	***x***	***y***	***z***	**$U_{\text{eq}}^{\text{a}}$ (Å^**2**^)**
Ba1A^b^	6*g*	0.176 (1)	1/2	1/2	0.019 (2)^c^
Ba1B^b^	6*g*	0.205 (1)	1/2	1/2	0.0192^c^
Ba2	4*e*	0.83409 (8)	*x*	*x*	0.0143 (3)
V1	12*i*	0.1577 (2)	*x*	0.3251 (2)	0.0102 (5)
V2	12*h*	0.1869 (2)	1/2	0	0.0092 (5)
Sb1	12*i*	0.21688 (5)	*x*	0.54212 (8)	0.0111 (2)
Sb2	12*i*	0.33244 (6)	*x*	0.01314 (8)	0.0096 (2)
Sb3	6*f*	0.3653 (1)	0	0	0.0085 (3)
Sb4	4*e*	0.11037 (8)	*x*	*x*	0.0112 (4)^c^
Sb5	4*e*	0.31655 (8)	*x*	*x*	0.0113 (4)
Sb6A^b^	1*b*	1/2	1/2	1/2	0.018 (3)
Sb7^b^	4*e*	0.459 (1)	*x*	*x*	0.0112^c^

^a^*U_eq_ is defined as one third of the trace of the orthogonalized U_ij_ tensor*.

b *Refined occupancies, occ(Ba1A) = 0.57(2), occ(Ba1B) = 1 – occ(Ba1A), occ(Sb6A) = 1 – occ(Ba1A), occ(Sb7) = 0.071(4)*.

c *Within the atomic pairs Ba1A and Ba1B and Sb4 and Sb7, respectively, anisotropic displacement parameters were constrained to be equal*.

**Table 4:** Atomic coordinates and equivalent isotropic displacement parameters for Ba_8_Ti_13−x_*M*_*x*_As_21_ (*M* = Nb, Ta).

**Atom**	**Site**	***x***	***y***	***z***	**$U_{\text{eq}}^{\text{a}}$ (Å^**2**^)**
**Ba**_**8**_**Ti**_**9.0(3)**_**Nb**_**4.0**_**As**_**21**_					
Ba1	32*f*	0.36972 (5)	*x*	*x*	0.0144 (4)
Ti/Nb1^b^	48*h*	0	0.1388 (1)	*y*	0.0116 (8)
Ti/Nb2^b^	4*a*	0	0	0	0.017 (2)
As1	32*f*	0.16332 (8)	*x*	*x*	0.0160 (6)
As2	24*e*	0.2306 (2)	0	0	0.0126 (6)
As3	24*d*	0	1/4	1/4	0.0126 (6)
As4	4*b*	1/2	1/2	1/2	0.015 (1)
**Ba**_**8**_**Ti**_**9.24(6)**_**Ta**_**3.76**_**As**_**21**_					
Ba1	32*f*	0.36964 (3)	*x*	*x*	0.0135 (3)
Ti/Ta1^c^	48*h*	0	0.13858 (5)	*y*	0.0122 (4)
Ti/Ta2^c^	4*a*	0	0	0	0.016 (2)
As1	32*f*	0.16325 (5)	*x*	*x*	0.0145 (4)
As2	24*e*	0.2306 (1)	0	0	0.0124 (4)
As3	24*d*	0	1/4	1/4	0.0123 (4)
As4	4*b*	1/2	1/2	1/2	0.0135 (9)

^a^*U_eq_ is defined as one third of the trace of the orthogonalized U_ij_ tensor*.

b *Ti/Nb1 = 0.73(2)Ti + 0.27Nb, Ti/Nb2 = 0.29(5)Ti + 0.71Nb*.

c *Ti/Ta1 = 0.703(4)Ti + 0.297Ta, Ti/Ta2 = 0.81(1)Ti + 0.19Ta*.

**Table 5:** Selected interatomic distances in Ba_5_V_12_*Pn*_19+x_ (*Pn* = As, Sb).

**Atoms**		**Distance** **(Å)**	
		**Ba_**5**_V_**12**_As_**19.02(1)**_**	**Ba_**5**_V_**12**_Sb_**19.36(2)**_**
Ba1A	—*Pn*1 × 4	3.2933 (9)	3.506 (2)
	—*Pn*2 × 2	3.318 (2)	3.485 (7)
	—*Pn*5 × 2	3.384 (2)	3.579 (6)
	—*Pn*2 × 2	3.484 (2)	3.675 (8)
	—*Pn*7 × 2	—	3.50 (1)
Ba1B	—*Pn*5 × 2	3.226 (8)	3.424 (5)
	—*Pn*1 × 4	3.262 (1)	3.4737 (9)
	—*Pn*6	3.33 (2)	3.58 (1)
	—*Pn*2 × 2	3.54 (1)	3.698 (8)
Ba2	—*Pn*7	3.294 (1)	—
	—*Pn*4 × 3	3.301 (1)	3.483 (2)
	—*Pn*2 × 3	3.323 (2)	3.587 (1)
	—*Pn*1 × 3	3.499 (2)	3.647 (2)
	—*Pn*3 × 3	3.567 (1)	3.734 (1)
V1	—*Pn*4	2.521 (3)	2.728 (3)
	—*Pn*5	2.531 (3)	2.726 (3)
	—*Pn*2 × 2	2.565 (1)	2.7513 (8)
	—*Pn*1	2.573 (3)	2.820 (3)
	—*Pn*3	2.600 (2)	2.748 (2)
	—V1 × 2	2.682 (4)	2.871 (4)
	—V2 × 2	2.720 (2)	2.877 (2)
V2	—*Pn*2 × 2	2.508 (1)	2.696 (2)
	—*Pn*1 × 2	2.508 (1)	2.7033 (8)
	—*Pn*3 × 2	2.618 (2)	2.793 (2)
	—V1 × 2	2.720 (2)	2.877 (2)
	—V2 × 2	3.078 (3)	3.205 (3)
*Pn*3	—*Pn*3	2.910 (4)	3.266 (3)
*Pn*5	—*Pn*1 × 3	2.965 (1)	3.2252 (9)
	—*Pn*7	—	2.98 (3)

**Table 6:** Selected interatomic distances in Ba_8_Ti_13−x_*M*_*x*_As_21_ (*M* = Nb, Ta).

**Atoms**		**Distance** **(Å)**	
		**Ba_**8**_Ti_**9.0(3)**_Nb_**4.0**_As_**21**_**	**Ba_**8**_Ti_**9.24(6)**_Ta_**3.76**_As_**21**_**
Ba1	—As1 × 3	3.319 (2)	3.320 (1)
	—As3 × 3	3.3506 (6)	3.3515 (3)
	—As4	3.539 (1)	3.5430 (9)
	—As2 × 3	3.621 (2)	3.623 (1)
Ti/*M*1	—As3	2.467 (2)	2.473 (1)
	—As2 × 2	2.610 (1)	2.6102 (9)
	—As1 × 2	2.619 (2)	2.620 (1)
	—Ti/*M*1 × 4	3.078 (2)	3.075 (1)
	—Ti/*M*2	3.078 (2)	3.075 (1)
Ti/*M*2	—Ti/*M*1 × 12	3.078 (2)	3.075 (1)
As1	—As3 × 3	3.203 (2)	3.2043 (8)

### First-Principle Calculations

For electronic structure calculations, a hypothetical disorder-free Ba_5_V_12_As_19_ model was employed. The split Ba1 site was treated as a single position with averaged atomic coordinates, and the partially occupied As6 and As7 sites were removed. An ordered model was also generated for Ba_8_Ti_9.0(3)_Nb_4.0_As_21_. For this purpose, the experimentally determined *Fm*$\bar{3}$*m* structure was transformed into a *I*4/*mmm* subgroup with half the original unit cell volume and the following transformation of the transition metal sites: 4*a* → 2*a*, 48*h* → 16*m* + 8*i*. The Nb atoms were placed exclusively in the 8*i* positions, resulting in the formula Ba_8_Ti_9_Nb_4_As_21_.

The calculations were executed with the TB-LMTO-ASA package (Jepsen and Andersen, 2000) at the local density approximation level applying the von Barth-Hedin exchange-correlation functional (von Barth and Hedin, 1972). Empty spheres were added to satisfy the Atomic Sphere Approximation (ASA). Chemical bonding was examined using Crystal Orbital Hamilton Population curves (COHP) (Steinberg and Dronskowski, 2018) and Electron Localization Function (ELF) (Savin et al., 1997), evaluated by the respective modules of the LMTO package. Electron density was integrated using the program Critic2 (Otero-de-la-Roza et al., 2014).

## Discussion

### Synthesis

The use of Pb as a flux for the synthesis of arsenides provides considerable benefits over the high-temperature annealing of the elements. High solubilities of many elements in molten Pb and its low melting point enable single crystal growth at moderately high temperatures, without significant losses of the highly volatile As. In addition, owing to their dilution, the dissolved starting materials in the course of a flux growth process display reduced chemical activity toward the reaction container, thereby preventing side reactions, which are often inevitable in the conventional high-temperature approach (Chen and Corbett, 2004; Baranets et al., 2018). A particular advantage of the flux approach is the possibility to produce single crystals containing elements with significantly different melting points, providing that all of the elements are reasonably soluble in the flux. We applied this approach to synthesize new multinary vanadium arsenides, since in this case, too, the high melting point of V (*T*_m_ = 2183 K) and the low sublimation point of As (*T*_subl_ = 883 K) make direct synthesis from the elements rather challenging.

Among the compositions in the *AE*–V–As–Pb systems (*AE* = Ca, Sr, Ba) used for single crystal growth attempts, only experiments with *AE* = Ba produced a new ternary phase, Ba_5_V_12_As_19+x_. For *AE* = Ca and Sr, binary arsenides were detected as products in all reactions. Although Ba_5_V_12_As_19+x_ can be obtained from different starting elemental ratios, so far it has been observed as a side product, indicating that further optimization of the procedure is necessary. In all cases, binary metal arsenides have been detected alongside Ba_5_V_12_As_19+x_. The low yield of Ba_5_V_12_As_19+x_is suggestive that the reaction conditions used for *AE* = Ca and Sr may also be improved to ultimately afford ternary compounds. Due to high reactivity of As toward various metals used as container materials in high-temperature reactions, systematic studies of the potential homogeneity range in Ba_5_V_12_As_19+x_ have been obstructed by side-reactions. However we would like to note that our investigations in the *AE*–Ti–Bi systems (*AE* = Sr, Ba), which host structurally related *AE*_5_Ti_12_Bi_19+x_ compounds, revealed that the Bi content depends on the alkaline-earth metal chosen, but the respective homogeneity ranges for a given *AE* were found to be rather narrow (Ovchinnikov and Bobev, 2018a).

The relatively low melting point of Sb (*T*_m_ = 903 K) allows its application as a reactive flux for the synthesis of antimonides. Such “self-flux” approach prevents potential contamination of the grown crystals by foreign elements. Crystal growth attempts in the Ba–V–Sb system yielded well-formed single crystals of Ba_5_V_12_Sb_19.36(2)_. It is worthwhile to mention that a very similar composition was found for the ternary Ba–V–Sb phase prepared by high-temperature annealing of an arc-melted sample with the nominal composition ≈ Ba5V12Sb25 (Failamani et al., 2015), suggesting that the obtained crystals are on the Sb-rich side of the homogeneity range. The crystal structure reported in Failamani et al. (2015) was refined as a Ba-deficient Ba_5−δ_V_12_Sb_19+x_. Our refinement does not provide evidence for any Ba vacancies but confirms the previously described splitting of the Ba site. Furthermore, this splitting is correlated with the disorder in the Sb substructure.

To date, flux growth experiments in the Sr–V–Sb system have resulted in binary antimonides only. In the Ca–V–Sb system, the exploratory work so far has resulted in the phase CaV_3_Sb_4_ (Ovchinnikov and Bobev, 2020), which is structurally unrelated to Ba_5_V_12_Sb_19+x_.

Crystal growth experiments in the *AE*–Ti–As–Pb systems (*AE* = Ca, Sr, Ba) always produced binary arsenides. Since the ternary compounds Ba_5_Ti_12_*Pn*_19+x_ (*Pn* = Sb, Bi), structurally related to Ba_5_V_12_*Pn*_19+x_ (*Pn* = As, Sb), have been synthesized and characterized in detail (Bie and Mar, 2009; Ovchinnikov and Bobev, 2018a; Han et al., 2019), it may seem surprising that the isoelectronic “Ba_5_Ti_12_As_19+x_” could not be obtained. To address this issue, we attempted the synthesis of this nominal composition in Nb and Ta tubes, as described in the Experimental section. Synthesis of arsenides in metal containers is often complicated by possible side reactions with the reactor walls. As a matter of fact, many new compounds were originally discovered as unexpected outcomes of such reactions (He et al., 2012; Baranets et al., 2018). Although the desired Ba_5_Ti_12_As_19+x_ was not detected in the products extracted from the Nb and Ta tubes after the high-temperature treatment, two isostructural compositions representing a new structure type were obtained – Ba_8_Ti_9.0(3)_Nb_4.0_As_21_ and Ba_8_Ti_9.24(6)_Ta_3.76_As_21_.

### Structure

*Ba*_5_*V*_12_*Pn*_19+*x*_ *(Pn* = *As, Sb)*. The crystal structures of Ba_5_V_12_As_19.02(1)_ and Ba_5_V_12_Sb_19.36(2)_ are closely related to each other and to those of the Ti-antimonide Ba_5_Ti_12_Sb_19+x_ (Bie and Mar, 2009) and Ti-bismuthide *AE*_5_Ti_12_Bi_19+x_ (*AE* = Sr, Ba) ternaries (Ovchinnikov and Bobev, 2018a). Subtle structural variations are associated with different realization of disorder in the *Pn* sites. Similarly to Ba_5_Ti_12_Sb_19+x_ and *AE*_5_Ti_12_Bi_19+x_ (*AE* = Sr, Ba), Ba_5_V_12_*Pn*_19+x_ (*Pn* = As, Sb) crystallize in the noncentrosymmetric space group *P*$\bar{4}$3*m*, with Pearson code *cP*72 (excluding the partially occupied sites). Refined Flack's parameter was close to zero for both compositions confirming correctness of the absolute configuration.

**Figure 1:**
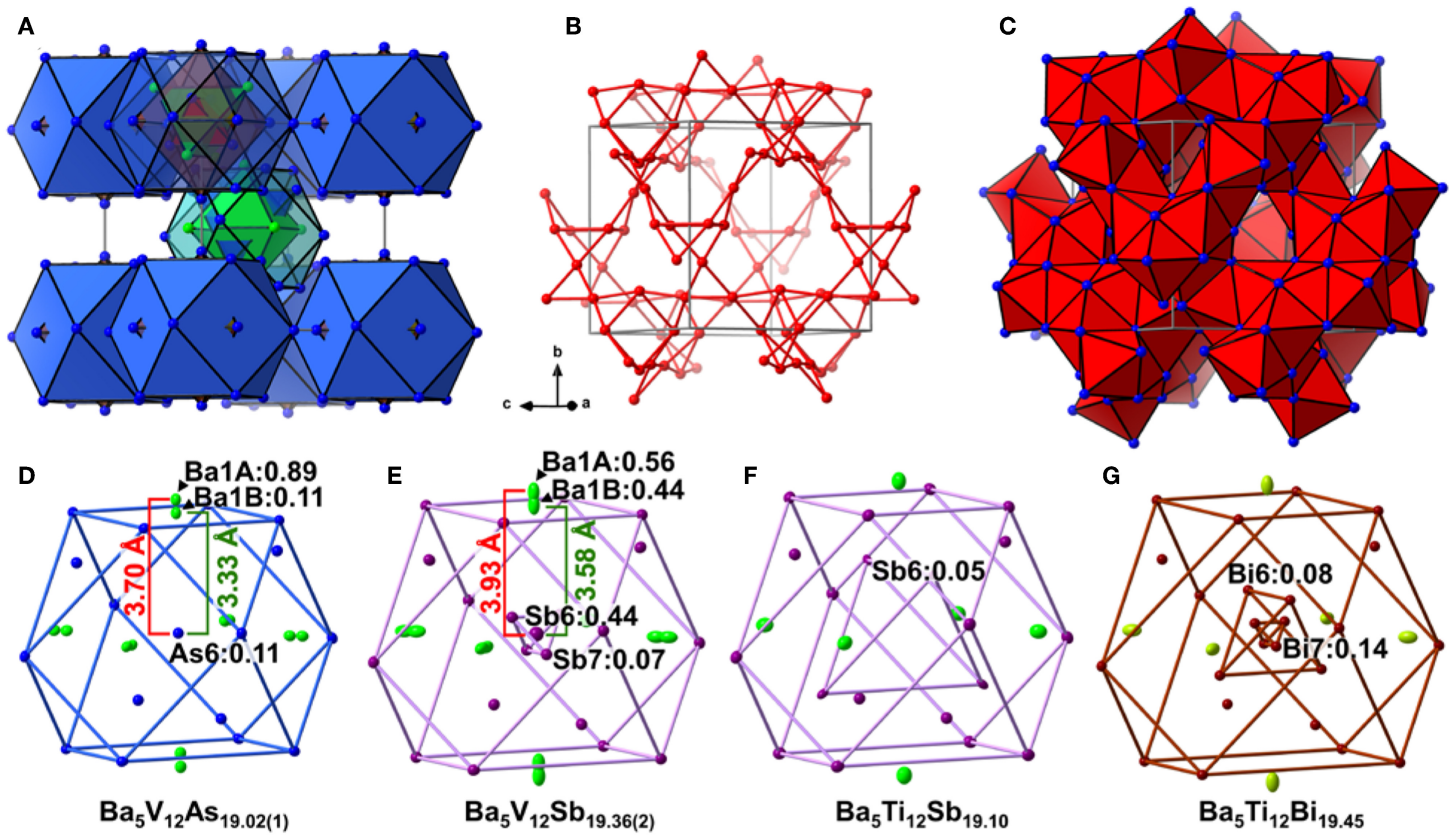
**(A)** Ba–*Pn* (*Pn* = As. Sb) substructure of Ba_5_V_12_*Pn*_19+x_ represented as a γ-brass-like cluster assembly. Ba and *Pn* atoms are depicted in green and blue, respectively. **(B)** Three-dimensional framework of the V atoms in the structure. The V–V contacts with distances ≤ 3.2 Å are shown. **(C)** Cage-like interlinking of the V*Pn*_6_ octahedra. The unit cell is outlined in gray. Close-up view of the central cuboctahedral cluster accommodating partially occupied *Pn* sites (*Pn* = As, Sb, Bi) in the structures of Ba_5_V_12_As_19.02(1)_ **(D)**, Ba_5_V_12_Sb_19.36(2)_ **(E)**, Ba_5_Ti_12_Sb_19.10_ **(F)**, Ba_5_Ti_12_Bi_19.45_ **(G)**. Partially occupied sites are indicated along with their occupancies. Thermal ellipsoids are drawn at the 50% probability level.

Analogously to the related Ti pnictides, in Ba_5_V_12_*Pn*_19+x_ (*Pn* = As, Sb), the deficiency-free part of the Ba–*Pn* substructure resembles a γ-brass-type framework (Figure 1A). The nested 26-atom cluster centered around the unit cell corner can be described as two interpenetrating Ba_4_ and *Pn*_4_ tetrahedra placed inside an As_6_ octahedron, which is in turn accommodated within an As_12_ cuboctahedron. The structure of the second nested polyhedron, located in the center of the unit cell, deviates from a regular γ-brass-type type cluster, and comprises an As_12_ cuboctahedron hosting a Ba_6_ octahedron overlapped with an As_4_ tetrahedron. The said Ba-*Pn* framework and a three-dimensional V scaffold are interpenetrated (Figure 1B). All V atoms are six-fold coordinated by the *Pn* atoms, and the resulting octahedra link by corner- and face-sharing and form a cage-like substructure (Figure 1C).

In all Ba_5_*M*_12_*Pn*_19+x_ structures (*M* = Ti, V; *Pn* = As, Sb, Bi), partially occupied *Pn* sites are present in the central cuboctahedral cluster. However, the disorder pattern is strongly dependent on the transition metal and the pnictogen. In Ba_5_V_12_As_19.02(1)_, extra As is located in the center of the unit cell (Figure 1D). The occupancy of this site refines to about 11 %. The adjacent Ba atoms in the corners of the octahedral shell show unphysically elongated thermal ellipsoids when refined as being positioned at a single crystallographic site. Splitting of this Ba position followed by independent refinement of the occupancies results in reasonable thermal parameters and occupation factors of about 89 and 11% for Ba1A and Ba1B, respectively. The splitting of Ba atom allows for two sets of distances to the center of the unit cell, where a partially occupied As atom (As6) is present −3.70 Å (Ba1A) and 3.33 Å (Ba1B), respectively. A good correlation of the Ba1B occupancy to that of the extra As site (As6) and the proximity of the total occupation of the two Ba atoms to unity implies the following account of the observed disorder: Whenever the As6 site is filled, the Ba_6_ octahedron “shrinks” to optimize the chemical interactions. Indeed, the Ba1B–As6 distance corresponds well to the typical Ba–As bonding contacts (He et al., 2010; Wang et al., 2011). When the center of the unit cell is empty, the Ba atoms in the octahedron move farther apart, likely due to electrostatic repulsion and optimization of the remaining Ba–As bonds.

A very similar picture is observed in Ba_5_V_12_Sb_19.36(2)_ (Figure 1E). In this case, the position in the center of the unit cell is 44% occupied by Sb (Sb6), and the corresponding splitting pattern of the adjacent Ba site indicates the same mechanism of structural relaxation to accommodate the extra Sb atom. In contrast to the arsenide, an additional Sb site (Sb7) is found close to the unit cell center at a distance of 0.87(2) Å, giving rise to a split position with an occupancy of around 7 %. Due to short distances to the partially occupied Sb6 and Ba1B sites, this position can only reside in the octahedron composed of the Ba1A atoms, with no Sb in its center.

Interestingly, the realization of disorder is different in the Ba_5_Ti_12_Sb_19+x_ and Ba_5_Ti_12_Bi_19+x_. In the former case (Figure 1F), only one partially occupied Sb site is present in the structure (Bie and Mar, 2009). This Sb position (Sb6) is filled only 5% of the time (for the experimentally determined composition Ba_5_Ti_12_Sb_19.10_) and is shifted from the center of the unit cell by 2.91 Å, forming a tetrahedron with an edge of 4.75 Å. Such a long interatomic separation renders any Sb6–Sb6 impossible. Yet a distance of 2.80 Å is observed between the Sb6 position and the Sb atoms on the triangular faces of the central cuboctahedral cluster, indicating covalent bonding. In Ba_5_Ti_12_Bi_19+x_(Figure 1G), there are two Bi sites with occupancies of about 8 and 14% (in Ba_5_Ti_12_Bi_19.45_), which are shifted from the center of the unit cell by 1.85 Å (Bi6) and 0.54 Å (Bi7), respectively (Ovchinnikov and Bobev, 2018a). The Bi6 atoms form a tetrahedron with an edge of 3.01 Å inside the central cuboctahedral cluster. Substitution studies provide evidence that the occupancy of this site can be increased up to 25% for the approximate composition SrEu_4_Ti_12_Bi_20_. This means that up to two Bi atoms can occupy the tetrahedron at a time, building a structural unit that is best viewed as a Bi_2_ dumbbell (Ovchinnikov and Bobev, 2018a).

To sum up, the Ba_5_*M*_12_*Pn*_19+x_ (*M* = Ti, V; *Pn* = As, Sb, Bi) compositions display very similar structures, but the subtle differences of the disorder pattern vary strongly with the transition metal and the pnictogen. The general trend which can be discerned from the side-by-side comparison of these structures, is the increasing degree of disorder upon going from the arsenides to the bismuthides. Apparently, the more compact structure of Ba_5_V_12_As_19.02(1)_ is not able to accommodate additional interstitial atoms. Another factor to be considered here is the lower stability of polyanions containing heavier pnictogens, due to the decrease of electronegativity in the order As–Sb–Bi (Ovchinnikov and Bobev, 2019d). The latter factor may call for additional stabilization by homoatomic bonding in antimonides and bismuthides, which is realized upon accommodating some extra pnictogen sites in the structure. Disregarding the disordered sites, the *Pn* atoms in Ba_5_*M*_12_*Pn*_19+x_ (*M* = Ti, V; *Pn* = As, Sb, Bi) may participate in hypervalent bonding, as suggested by some relatively short *Pn*–*Pn* contacts, e.g., 2.910(4) Å and 2.965(1) Å in Ba_5_V_12_As_19.02(1)_, and 3.266(3) Å and 3.2252(9) Å in Ba_5_V_12_Sb_19.36(2)_.

*Ba*_8_*Ti*_13−*x*_*M*_*x*_*As*_21_ *(M* = *Nb, Ta)*. The isostructural Ba_8_Ti_13−x_Nb_*x*_As_21_ and Ba_8_Ti_13−x_Ta_*x*_As_21_ adopt a new structure type with space group *Fm*$\bar{3}$*m* and Pearson symbol *cF*168. The serendipitous incorporation of Nb and Ta metal from the crucible material into the structure, adds to many other instances of arsenides in particular (He et al., 2012; Baranets et al., 2018), where these typically high-melting and hard to activate elements prove to be quite reactive.

**Figure 2:**
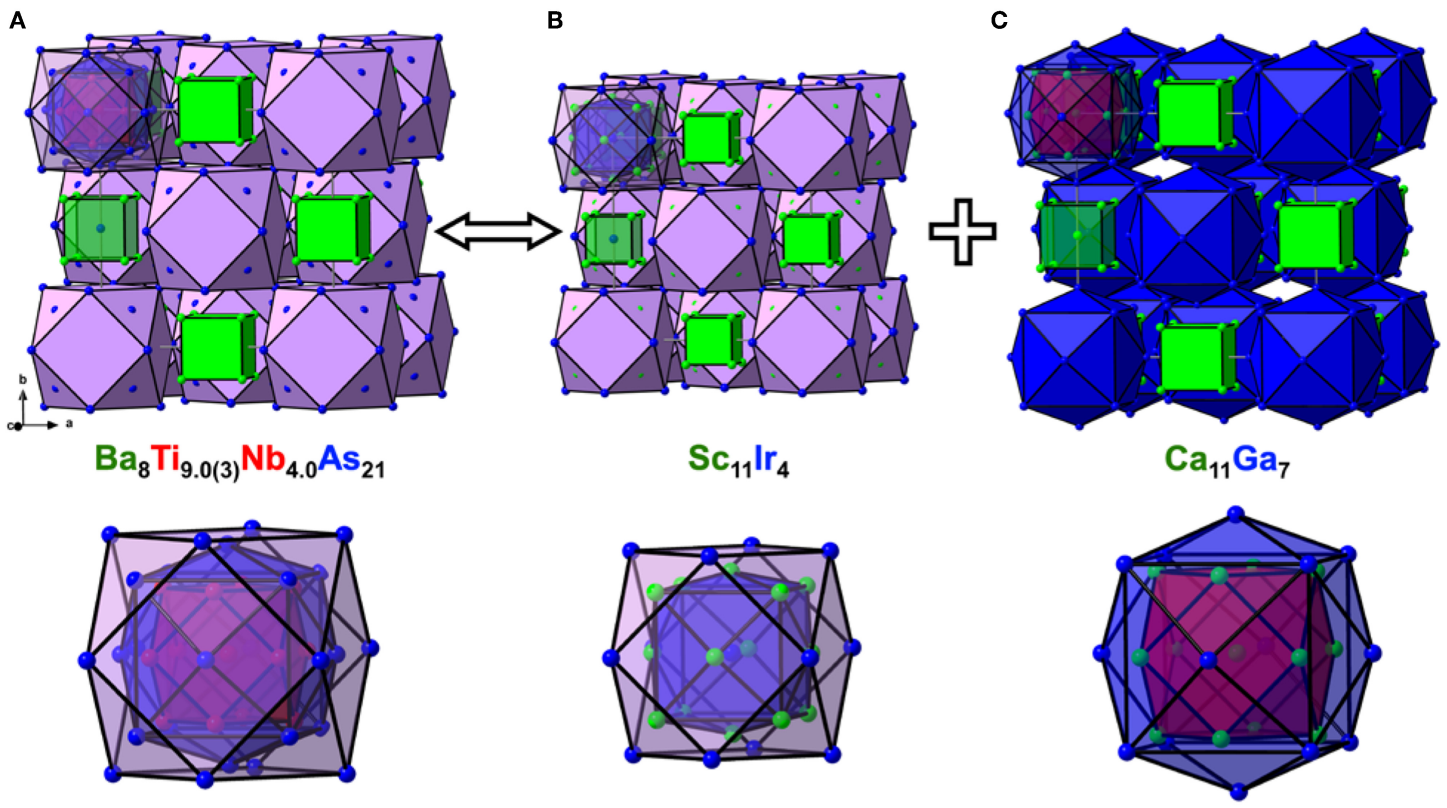
Crystal structures of Ba_8_Ti_9.0(3)_Nb_4.0_As_21_ **(A)**, Sc_11_Ir_4_ **(B)**, and Ca_11_Ga_7_ **(C)** in cluster representation, drawn at the same scale, along with the close-up view of the respective nested polyhedral clusters at the bottom. The shell structure of the nested and centered clusters is highlighted.

There are two symmetry-independent sites in the presented structures where the targeted transition metal atoms, Ti, and the atoms of group 5 element are found to be statistically mixed. These sites, hereafter referred to as *TM*1 and *TM*2 for brevity, are located in the vertices of a cuboctahedron and in its center, respectively, and account for isolated *TM*_13_ metal clusters (Figure 2A). The *TM*1–*TM*1 and *TM*1–*TM*2 distances are equal and measure *d*_*TM*__−TM_ = 3.078(2) Å and 3.075(1) Å for the Nb and Ta structures, respectively. The *TM*_13_ clusters can therefore be described as fragments of a perfect *fcc*-packing. The occurrence of such units is at first glance surprising, since the constituting transition metals form hexagonal close-packed (Ti) or *bcc* (Nb, Ta) structures at ambient conditions. A detailed account of the electronic interactions in the clusters will be given in the discussion of the electronic structure and chemical bonding (see below).

To simplify the description of the complex atomic arrangement in Ba_8_*TM*_13_As_21_ and highlight relationships with other cubic metal-rich compounds, it is convenient to represent the structure as a packing of polyhedra, in a similar manner as was previously described for Ba_5_V_12_*Pn*_19+x_. The *TM*1 sites are five-fold coordinated by As atoms, with the *TM*2 site completing a pseudo-octahedral coordination. The *TM*–As distances are very similar in both compounds and fall in the range 2.47–2.62 Å, in good agreement with the literature data on other titanium arsenides (Bachmayer et al., 1955; Nuss et al., 1996; Lee et al., 2001). The basal As atoms of the pseudo-octahedra are located in the vertices of an As_14_ tetrakis hexahedron around the central *TM*2 atom, whereas the apical As atoms can be viewed as forming a regular As_12_ cuboctahedron around the same center. The described geometrical construction yields a nested four-shell polyhedron, [*TM*2@*TM*1_12_@As_14_@As_12/2_] (= *TM*_13_As_20_), where the innermost “shell” is built up of a single atom. The notation “As_12/2_” reflects the fact that each of the 12 As atoms in the outer cuboctahedral shell is shared between two symmetrically equivalent polyhedra. The shortest As–As distance in the structure is observed between the As atoms in the outermost and penultimate shells of the nested polyhedron and is found to be 3.203(2) Å and 3.2043(8) Å for the Nb and Ta representatives, respectively. The four-shell units form an *fcc* arrangement with the octahedral voids occupied by isolated As-centered [AsBa_8_] cubes.

The presented polyhedral description suggests that the structure of Ba_8_*TM*_13_As_21_ can be thought of as a combination of the Sc_11_Ir_4_ (Figure 2B) and Ca_11_Ga_7_ (Figure 2C) structure types (Villars and Calvert, 1991). All three structures adopt space group *Fm*$\bar{3}$*m* but show different populations of the Wyckoff sites. The nested polyhedron in Sc_11_Ir_4_, expressed as [Ir@Sc_14_@Ir_12/2_], lacks the inner cuboctahedral shell, hosted by the similar unit in Ba_8_*TM*_13_As_21_. The larger polyhedron in Ca_11_Ga_7_, on the contrary, accommodates a cuboctahedron, but represents a “peeled” version of the four-shelled cluster in the arsenide structure, with the following sequence of the shells: [Ca@Ca_12_@Ga_14_]. In both Sc_11_Ir_4_ and Ca_11_Ga_7_, centered cubes occupy the octahedral cavities in the *fcc* packing of the nested polyhedra.

The tetrahedral voids remain unoccupied in all three structures discussed above. Interestingly, in Ba_8_*TM*_13_As_21_, the distance between the center of the tetrahedral hole to the nearest As atoms measures 2.355(2) Å and 2.358(1) Å in the Nb and Ta representatives, respectively. These numbers imply that it might be possible to intercalate a small metal atom into this cavity, which would result in a reasonable tetrahedral coordination and could change/augment the valence electron count, as discussed in the next section.

### Electronic Structure

**Figure 3:**
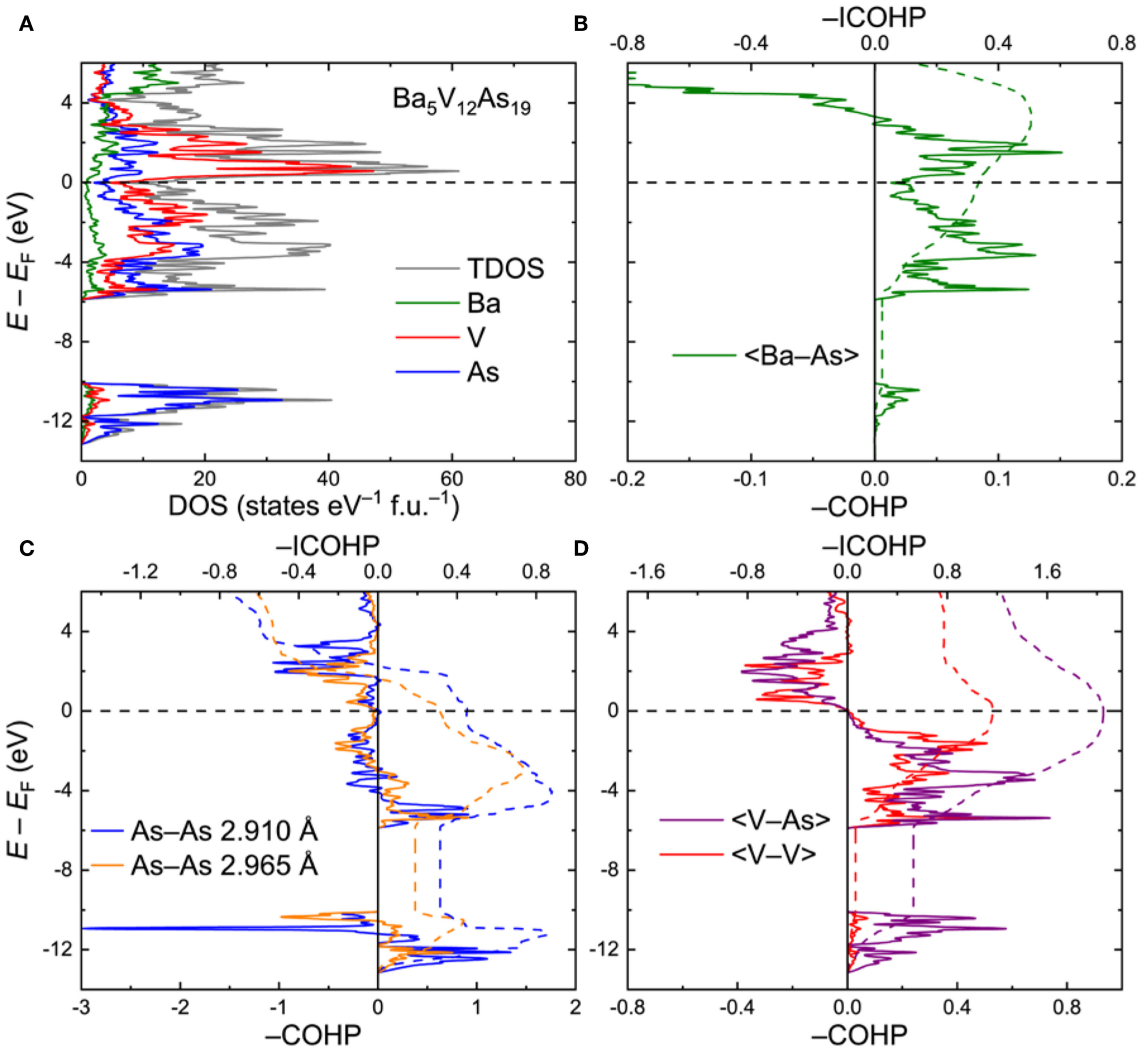
**(A)** Total and projected electronic densities of states (DOS) for Ba_5_V_12_As_19_. **(B–D)** Crystal Orbital Hamilton Population curves (COHP) for selected contacts. Dashed lines denote integrated COHP curves.

*Ba*_5_*V*_12_*As*_19_. To interrogate the electronic structure and chemical bonding in the Ba_5_V_12_*Pn*_19+x_ compounds, an idealized ordered Ba_5_V_12_As_19_ model was utilized. Total and projected electronic densities of states (DOS) are shown in Figure 3A. The Fermi level (*E*_F_) is located in the vicinity of a dip in the DOS: The electronic states close to *E*_F_ are primarily composed of the hybridized V(3d) and As(4p) orbitals. The Ba–As bonding deviates significantly from a simple ionic character as indicated by the sizeable continuous contribution of the Ba electronic states around the Fermi level. The As(4s) orbitals are highly localized in a narrow energy interval around *E* – *E*_F_ ≈ −11 eV. These states mainly manifest the presence of the As lone pairs in the structure.

Crystal Orbital Hamilton Population graphs (COHP) for average selected interatomic interactions are plotted in Figures 3B–D. In line with the conclusion derived from the projected DOS, the Ba–As interactions do not display a typical ionic nature. The bonding is underoptimized at *E*_F_, with an energy window of unoccupied bonding states extending up to *E* – *E*_F_ ≈ 2.9 eV (Figure 3B). The negative integrated COHP (–COHP) for the Ba–As amounts to about 0.34 eV/bond on average.

The two shortest As–As contacts demonstrate similar features in their COHP plots (Figure 3C). Both contacts are characterized by a combination of bonding and antibonding states below *E*_F_. The resulting attractive interaction is rather weak, yet the –ICOHP values of 0.31 and 0.45 eV/bond for the longer and shorter As–As contacts, respectively, are comparable with the numbers found for the Ba–As bonds. Altogether, the observed COHP pattern for the As–As contacts is in accordance with hypervalent (electron-rich) chemical bonding (Papoian and Hoffmann, 2000; Ovchinnikov and Bobev, 2018b,c).

In contrast, the V–As and V–V bonds are optimized at the Fermi level, with the respective average –ICOHP magnitudes of 2.05 and 1.16 eV/bond (Figure 3D). It is worth noting that in Ba_5_V_12_As_19_, only a small number of V–As and V–V bonding states are available in the near vicinity of *E*_F_ down to *E* – *E*_F_ ≈ −1.2 eV. This energy may correspond to the lowest electron count which would retain the structural stability. Indeed, upon lowering the Fermi level by more than 1.2 eV, the bonding V–As and V–V states get progressively vacated, perturbing the respective bonding. Introduction of small amounts of extra As, as in the experimentally determined composition Ba_5_V_12_As_19.02(1)_, can be viewed as a mild oxidation of the discussed Ba_5_V_12_As_19_ composition and will have a very small effect on the V–As and V–V bonds. Furthermore, the hitherto unknown Ba_5_Ti_12_As_19_ would also fall in the stability region according to the suggested electronic considerations. If a rigid band model can be applied in this case, the electronic structure of Ba_5_Ti_12_As_19_ can be derived from that of Ba_5_V_12_As_19_ by shifting the Fermi level down by about 0.89 eV, which would correspond to removal of 12 electrons per formula unit with respect to the vanadium composition. In fact, the resulting hypothetical electronic structure would tolerate even further oxidation, e.g., by incorporation of extra pnictogen atoms. This prediction suggests that the apparent non-existence (or lower stability) of Ba_5_Ti_12_As_19+x_does not originate from electronic reasons. Atomic size factors or peculiarities of the reaction pathways in the Ba–Ti–As system should be examined in detail to resolve this conundrum.

**Figure 4:**
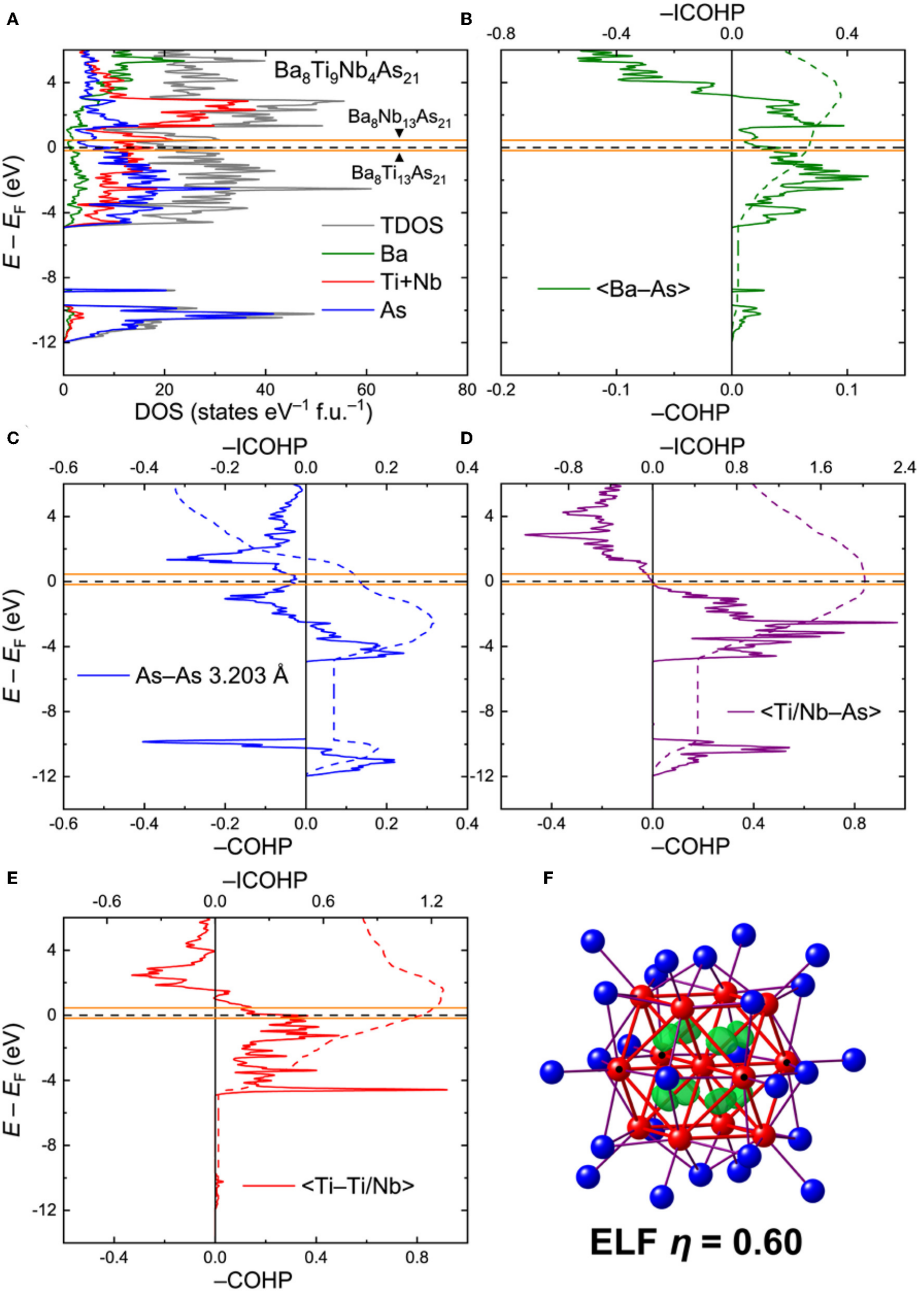
**(A)** Total and projected electronic densities of states (DOS) for Ba_8_Ti_9_Nb_4_As_21_. **(B–E)** Crystal Orbital Hamilton Population curves (COHP) for selected contacts. Positions of the Fermi level for the hypothetical ternary compositions Ba_8_Ti_13_As_21_ and Ba_8_Nb_13_As_21_ are given in orange. Dashed lines denote integrated COHP curves. **(F)** Isosurface of the Electron Localization Function (η = 0.60) inside the cuboctahedral transition metal cluster. Location of the Nb atoms in the ordered model is indicated with black dots.

*Ba*_8_*Ti*_9_*Nb*_4_*As*_21_. For analysis of the electronic interactions in the Ba_8_Ti_13−x_*M*_*x*_As_21_ phases (*M* = Nb, Ta), an ordered model with composition Ba_8_Ti_9_Nb_4_As_21_ was generated. The electronic density of states (DOS) for this model is given in Figure 4A. In contrast to the Ba_5_V_12_As_19_ case, the Fermi level in Ba_8_Ti_9_Nb_4_As_21_ crosses a peak in the DOS, mainly composed of the transition metal d orbitals. The high density of states may indicate some electronic instabilities, warranting further experimental studies. In this respect, it is worthwhile to note that spin-polarized calculations did not indicate any localized magnetism, rendering magnetic instability quite improbable.

The calculated COHP curves for averaged selected contacts in the structure are presented in Figures 4B–E. For the sake of simplicity, the contacts involving Ti and Nb in the ordered model were averaged. The Ba–As interactions bear resemblance to the respective contacts in Ba_5_V_12_As_19_, with somewhat underoptimized bonding at *E*_F_ (Figure 4B). The average –ICOHP value for these contacts is 0.27 eV/bond.

The shortest As–As contact, despite a considerable interatomic separation, displays a non-negligible, yet underoptimized, bonding interaction, with a domain of occupied antibonding states just below *E*_F_ (Figure 4C), akin to Ba_5_V_12_As_19_. In an idealized picture, these bonds can be treated as one-electron interactions (Papoian and Hoffmann, 2000). This very crude approximation allows assignment of formal charges to the As sites. An isolated As atom (i.e., with no short As–As contacts) has a formal charge of 3–, whereas every one-electron bond lowers the absolute negative charge by 0.5. In terms of such one-electron bonds, the As atoms in Ba_8_Ti_9_Nb_4_As_21_ are either isolated or three- and four-fold coordinated. The schematic representation of the As framework is shown in Figure S1. In the limit of full electron localization, the formula can be expressed as (Ba^2+^)_8_[Ti_9_Nb_4_]^23+^(As^3−^)_7_(As^1.5−^)_8_(As^1−^)_6_. Although this electronic distribution is an oversimplification, it will provide an insight into the metal-metal bonding in the clusters, as discussed below.

Quite notably, the transition-metal–arsenic bonds are perfectly optimized at the Fermi level (Figure 4D). The strong covalent nature of these bonds is evident from the high average—ICOHP magnitude of 2.02 eV/bond. Similarly to Ba_5_V_12_As_19_, the number of the occupied bonding states just below *E*_F_ and the vacant antibonding states close to *E*_F_ is rather small, suggesting that moderate changes in the electron count would not strongly affect the bonding. In particular, shifting the Fermi level down by 0.17 eV or lifting it up by 0.45 eV, corresponding to the positions of *E*_F_ in the hypothetical Ba_8_Ti_13_As_21_ and Ba_8_Nb_13_As_21_, respectively (orange lines in Figure 4), will have virtually no influence on the transition-metal–arsenic interactions.

The situation is different for the metal–metal bonds, which appear to be underoptimized for the composition Ba_8_Ti_9_Nb_4_As_21_, owing to the availability of unoccupied bonding states above *E*_F_ (Figure 4E). These states extend up to *E* – *E*_F_ ≈ 1.54 eV, which would correspond to ~35 extra electrons per formula unit. This finding shows that electron doping in Ba_8_Ti_9_Nb_4_As_21_ is favorable and expected to enhance the metal-metal interactions, which are already quite strong for the examined composition, with an average –ICOHP of 1.14 eV/bond. The maximum –ICOHP value that can be achieved by filling up all the bonding states above *E*_F_ amounts to 1.26 eV/bond. At this high electron count, however, some occupation of the antibonding transition-metal–arsenic states will be attained. Since the structure is dominated by the latter kind of bonds in terms of their number and relative strength, it is likely that these bonds will be pivotal for the overall stability.

Analysis of the Electron Localization Function (ELF) for the Ba–As contacts did not reveal any localization maxima but indicated small deviation of the ELF distribution from a spherical shape around the atoms, typical for highly polar bonds. A much more pronounced deviation was found for the *TM*–As contacts (Figure S2a). Increased values and flattening of the ELF was also observed for the short As–As contacts (Figure S2b). Clear localization domains are visible inside the tetrahedral voids of the cuboctahedral cluster, suggesting multi-center metal–metal bonding (Figure 4F). Detailed inspection of the ELF sections revealed the presence of additional ELF maxima on the lines connecting the central transition metal atom to the twelve vertices of the cuboctahedron, indicating two-center bonds (Figure S2c). In a very naïve picture of perfect electron localization, the formation of eight multi-center and 12 two-center bonds would require 40 electrons in total, if all these bonds are treated as two-electron. By considering the formula derived above, (Ba^2+^)_8_[Ti_9_Nb_4_]^23+^(As^3−^)_7_(As^1.5−^)_8_(As^1−^)_6_, the total number of valence electrons inside the [Ti_9_Nb_4_] cluster is 9 × 4 + 4 × 5 – 23 = 33. The limiting hypothetical compositions Ba_8_Ti_13_As_21_ and Ba_8_Nb_13_As_21_ would correspond to 29 and 42 cluster electrons, respectively. The proposed simple electron accounting is in line with the conclusion that electron doping of Ba_8_Ti_9_Nb_4_As_21_ will stabilize the bonding inside the clusters. A more accurate approach of determining the number of cluster electrons is direct integration of electron density inside the Bader basins of the transition metal atoms. This method yields 37.92 electrons in the [Ti_9_Nb_4_] cluster, which is somewhat below the optimal number obtained by the overly-simplified electron counting above. Since the bonding in the metallic Ba_8_Ti_9_Nb_4_As_21_ is rather delocalized, the valence electron considerations detailed above should be deemed a *qualitative* rationale for the stability of the discussed complex crystal structure. It seems very likely that Ba_8_Ti_9_Nb_4_As_21_ will show some flexibility with respect to the total electron count and definitely requires a more detailed experimental examination.

## Conclusions

The metal flux method is a powerful approach for crystal growth of various kinds of intermetallic compounds. Its simple design and wide applicability makes it an efficient tool for exploratory synthetic research. Two ternary barium vanadium pnictides, Ba_5_V_12_As_19.02(1)_ and Ba_5_V_12_Sb_19.36(2)_ were successfully grown as mm-sized single crystals employing selected flux materials—Pb and Sb, respectively. The crystal structure of both compounds can be described as γ-brass-like cluster assemblies based on Ba and *Pn* (*Pn* = As, Sb), interpenetrated by three-dimensional V scaffolds. The observed non-stoichiometry results from additional partially occupied *Pn* positions, which show different disorder patterns depending on the pnictogen. First-principle calculations predict high flexibility of the structure with respect to the electron count, which explains the existence of the related electron-poorer Ti phases Ba_5_Ti_12_Sb_19+x_ and *AE*_5_Ti_12_Bi_19+x_ (*AE* = Sr, Ba). Following the analogy between the V and Ti pnictides, we attempted to obtain the hitherto unknown Ba_5_Ti_12_As_19+x_, employing the flux technique and conventional high-temperature annealing of the elements in metal tubes. The latter approach yielded two new compounds, Ba_8_Ti_9.0(3)_Nb_4.0_As_21_ and Ba_8_Ti_9.24(6)_Ta_3.76_As_21_, as a result of side-reactions with the container material. The quaternary compositions crystallize isotypically in a new structure type, which displays isolated *fcc*-type clusters composed of statistically mixed Ti and *M* atoms (*M* = Nb, Ta). First-principle calculations reveal that the bonding within these units displays two-center and multi-center features and complex electron distribution. The presented examples demonstrate that the field of early transition metal pnictides deserves additional study, as many new compounds with potentially interesting structures and properties are likely to be found. In this respect, the flux method proves to be a handy tool for exploration of these systems.

## Data Availability Statement

The datasets generated for this study can be found in the The Cambridge Crystallographic Data Centre (CCDC) under the deposition numbers 1959265-1959268.

## Author Contributions

AO designed and carried out the synthesis, performed crystallographic characterization and first-principle calculations, and prepared the initial draft of the manuscript. SB supervised the project and finalized the manuscript.

### Conflict of Interest

The authors declare that the research was conducted in the absence of any commercial or financial relationships that could be construed as a potential conflict of interest.

## Footnotes

^1^During the refinement of the Ba_5_V_12_As_19.02(1)_ structure, a considerable residual electron density was detected in the corners of the unit cell. Modifications of the absorption correction did not result in disappearance of this feature. The only chemical species which would show reasonable local coordination when placed in this position was As. After ascribing the observed density peak to As (labeled here As7), the resulting Ba–As contacts were within the distance range found for other bonds of this sort in the structure. The refined occupancy of As7 was only about 2 %. Owing to an unphysically short As7–As4 distance of 2.217(1) Å, the occupancy of As4 was set to 100 – occupancy(As7) %. Acknowledging the possibility that the observed residual density peak is an artifact of the special position, we abstain from the discussion of this disorder until additional data are available. Due to the very small occupancy, the effect of the As7 site on the structure and composition is negligible.

## References

[B1] Bachmayer K. Nowotny H. Kohl A. (1955). Die Struktur von TiAs. Monatsh. Chem. 86, 39–43. 10.1007/BF00899271

[B2] Bao J.-K. Bugaris D. E. Zheng H. Willa K. Welp U. Chung D. Y. (2019). Superconductivity in Y_7_Ru_4_InGe_12_. Phys Rev Mater. 3:024802 10.1103/PhysRevMaterials.3.024802

[B3] Bao J.-K. Liu J.-Y. Ma C.-W. Meng Z.-H. Tang Z.-T. Sun Y.-L. (2015). Superconductivity in quasi-one-dimensional K_2_Cr_3_As_3_ with significant electron correlations. Phys. Rev. X. 5:011013 10.1103/PhysRevX.5.011013

[B4] Baranets S. Bobev S. (2019). From the ternary phase Ca_14_Zn_1+δ_Sb_11_ (δ ≈ 0.4) to the quaternary solid solutions Ca_14−x_*RE*_*x*_ZnSb_11_ (*RE* = La–Nd, Sm, Gd, *x* ≈ 0.9). A tale of electron doping via rare-earth metal substitutions and the concomitant structural transformations. Inorg. Chem. 58, 8506–8516. 10.1021/acs.inorgchem.9b00809 31194532

[B5] Baranets S. Darone G. M. Bobev S. (2019a). Synthesis and structure of Sr_14_Zn_1+x_As_11_ and Eu_14_Zn_1+x_As_11_ (*x* ≤ 0.5). New members of the family of pnictides isotypic with Ca_14_AlSb_11_, exhibiting a new type of structural disorder. J. Solid. State. Chem. 280:120990 10.1016/j.jssc.2019.120990

[B6] Baranets S. He H. Bobev S. (2018). Niobium-bearing arsenides and germanides from elemental mixtures not involving niobium: a new twist to an old problem in solid-state synthesis. Acta. Crystallogr. C. 74, 623–627. 10.1107/S2053229618005739 29726473

[B7] Baranets S. Voss L. Stoyko S. Bobev S. (2019b). Synthesis, crystal structure and physical properties of the solid solutions Ca_14−x_*RE*_*x*_CdSb_11_ (*RE* = La–Nd, Sm, Gd–Yb, *x* ≈ 0.85 ± 0.15). J. Appl. Phys. 125:245101 10.1063/1.5099632

[B8] Bie H. Mar A. (2009). Ba_5_Ti_12_Sb_19+x_, a polar intermetallic compound with a stuffed γ-brass structure. J. Solid. State. Chem. 182, 3131–3137. 10.1016/j.jssc.2009.08.030

[B9] Brylak M. Jeitschko W. (1994). U_3_TiSb_5_, U_3_VSb_5_, U_3_CrSb_5_, and U_3_MnSb_5_ with ‘Anti'-Hf_5_Sn_3_Cu Type Structure. Z Naturforsch. B. 49, 747–752. 10.1515/znb-1994-0605

[B10] Canfield P. C. Fisk Z. (1992). Growth of single crystals from metallic fluxes. Philos. Mag. B. 65, 1117–1123. 10.1080/13642819208215073

[B11] Chen L. Corbett J. D. (2004). R_6_TT‘_2_, New variants of the Fe_2_P structure type. Sc_6_TTe_2_ (T = Ru, Os, Rh, Ir), Lu_6_MoSb_2_, and the anti-typic Sc_6_Te_0.80_Bi_1.68_. Inorg. Chem. 43, 436–442. 10.1021/ic0302581 14731005

[B12] Childs A. B. Baranets S. Bobev S. (2019). Five new ternary indium-arsenides discovered. Synthesis and structural characterization of the Zintl phases Sr_3_In_2_As_4_, Ba_3_In_2_As_4_, Eu_3_In_2_As_4_, Sr_5_In_2_As_6_ and Eu_5_In_2_As_6_. J. Solid. State. Chem. 278:120889 10.1016/j.jssc.2019.07.050

[B13] Doan P. Gooch M. Tang Z. Lorenz B. Möller A. Tapp J. (2012). Ba_1−x_Na_*x*_Ti_2_Sb_2_O(0.0 ≤ *x* ≤ 0.33): a layered titanium-based pnictide oxide superconductor. J. Am. Chem. Soc. 134, 16520–16523. 10.1021/ja3078889 22998020

[B14] Failamani F. Grytsiv A. Giester G. Polt G. Heinrich P. Michor H.. (2015). Ba_5_{V,Nb}_12_Sb_19+x_, novel variants of the Ba_5_Ti_12_Sb_19+x_ -type: crystal structure and physical properties. Phys. Chem. Chem. Phys. 17, 24248–24261. 10.1039/C5CP04000K 26327293

[B15] Felder J. B. Weiland A. Hodovanets H. McCandless G. T. Estrada T. G. Martin T. J.. (2019). Law and disorder: special stacking units—building the intergrowth Ce_6_Co_5_Ge_16_. Inorg. Chem. 58, 6037–6043. 10.1021/acs.inorgchem.9b00350 31009213

[B16] Gelato L. M. Parthé E. (1987). *STRUCTURE TIDY* – a computer program to standardize crystal structure data. J. Appl. Crystallogr. 20, 139–143. 10.1107/S0021889887086965

[B17] Han F. Bao J.-K. Malliakas C. D. Sturza M. Du Y. Chung D. Y. (2019). Enormous electron-electron scattering in the filled-cage cubic compound Ba_10_Ti_24_Bi_39_. Phys. Rev. Mater. 3:105001 10.1103/PhysRevMaterials.3.105001

[B18] He H. Stearrett R. Nowak E. R. Bobev S. (2010). BaGa_2_*Pn*_2_ (*Pn* = P, As): New semiconducting phosphides and arsenides with layered structures. Inorg. Chem. 49, 7935–7940. 10.1021/ic100940b 20701248

[B19] He H. Tyson C. Bobev S. (2012). Synthesis and crystal structures of the quaternary zintl phases RbNa_8_Ga_3_*Pn*_6_ (*Pn* = P, As) and Na_10_NbGaAs_6_. Crystals 2, 213–223. 10.3390/cryst2020213

[B20] Jayasinghe A. S. Lai Y. Baumbach R. Latturner S. E. (2019). U_1.33_*T*_4_Al_8_Si_2_ (*T* = Ni, Co): complex uranium silicides grown from aluminum/gallium flux mixtures. Inorg. Chem. 58, 12209–12217. 10.1021/acs.inorgchem.9b01627 31454237

[B21] Jepsen O. Andersen O. K. (2000). The Stuttgart TB-LMTO-ASA Program, Version 4.7; Max-Planck-Institut für Festkörperforschung. Stuttgart.

[B22] Kanatzidis M. G. Pöttgen R. Jeitschko W. (2005). The metal flux: a preparative tool for the exploration of intermetallic compounds. Angew. Chem. Int. Ed. 44, 6996–7023. 10.1002/anie.200462170 16259022

[B23] Khan M. A. McCandless G. T. Benavides K. A. Martin T. J. Palacios A. M. Samuel A. W. B. (2018). Crystal Growth and Magnetic Properties of Pr_3_Co_2+x_Ge_7_ and the Sn-Stabilized Ln_3_Co_2+x_Ge_7−y_Sn_*y*_ (Ln = Pr, Nd, Sm). Cryst. Growth. Des. 18, 6028–6034. 10.1021/acs.cgd.8b00868

[B24] Latturner S. E. (2018). Clusters, assemble: growth of intermetallic compounds from metal flux reactions. Acc. Chem. Res. 51, 40–48. 10.1021/acs.accounts.7b00483 29257668

[B25] Lee C. S. Dashjav E. Kleinke H. (2001). Structure prediction using our semiempirical structure map: the crystal structure of the new arsenide ZrTiAs. Chem. Mater. 13, 4053–4057. 10.1021/cm010433g

[B26] Ma X. Chen B. Latturner S. E. (2012). Synthesis and properties of new multinary silicides R_5_Mg_5_Fe_4_Al_*x*_Si_18−x_ (R = Gd, Dy, Y, *x* ≈ 12) grown in Mg/Al Flux. Inorg. Chem. 51, 6089–6095. 10.1021/ic202735b 22591197

[B27] Nakamura S. Kano T. Ohara S. (2019). Magnetic ordering in kondo lattice compound YbIr_3_Si_7_. J. Phys. Soc. Jpn. 88:093705 10.7566/JPSJ.88.093705

[B28] Nuss J. Hönle W. Peters K. Schnering H. G. V. (1996). Tetrapnictidotitanate(IV) M_4_TiX_4_ (M = Sr, Ba; X = P, As), hierarchische Derivate der KGe-Struktur K_4_□Ge_4_. Z. Anorg. Allg. Chem. 622, 1879–1885. 10.1002/zaac.19966221112

[B29] Otero-de-la-Roza A. Johnson E. R. Luaña V. (2014). Critic2: a program for real-space analysis of quantum chemical interactions in solids. Comput. Phys. Commun. 185, 1007–1018. 10.1016/j.cpc.2013.10.026

[B30] Ovchinnikov A. Bobev S. (2018a). Synthesis, crystal and electronic structure of the titanium bismuthides Sr_5_Ti_12_Bi_19+x_, Ba_5_Ti_12_Bi_19+x_, and Sr_5−δ_ Eu_δ_Ti_12_Bi_19+x_ (*x* ≈ 0.5–1.0; δ ≈ 2.4, 4.0). Eur. J. Inorg. Chem. 2018, 1266–1274. 10.1002/ejic.201701426

[B31] Ovchinnikov A. Bobev S. (2018b). On the effect of Ga and In substitutions in the Ca_11_Bi_10_ and Yb_11_Bi_10_ bismuthides crystallizing in the tetragonal Ho_11_Ge_10_ structure type. Acta. Crystallogr. C. 74, 269–273. 10.1107/S2053229618001596 29504553

[B32] Ovchinnikov A. Bobev S. (2018c). Undistorted linear Bi chains with hypervalent bonding in La_3_TiBi_5_ from single-crystal X-ray diffraction. Acta. Crystallogr. C. 74, 618–622. 10.1107/S205322961800565X 29726472

[B33] Ovchinnikov A. Bobev S. (2019a). Multifaceted Sn–Sn bonding in the solid state. Synthesis and structural characterization of four new Ca–Li–Sn compounds. Dalton. Trans. 48, 14398–14407. 10.1039/C9DT02803J 31509139

[B34] Ovchinnikov A. Bobev S. (2019b). Synthesis, and crystal and electronic structures, of the titanium-rich bismuthides *AE*_3_Ti_8_Bi_10_ (*AE* = Sr, Ba, Eu). Inorg. Chem. 58, 2934–2941. 10.1021/acs.inorgchem.8b01952 30212194

[B35] Ovchinnikov A. Bobev S. (2019c). Layered quaternary germanides—synthesis and crystal and electronic structures of *AE*Li_2_In_2_Ge_2_ (*AE* = Sr, Ba, Eu). Inorg. Chem. 58, 7895–7904. 10.1021/acs.inorgchem.9b00588 31140792

[B36] Ovchinnikov A. Bobev S. (2019d). Zintl phases with group 15 elements and the transition metals: a brief overview of pnictides with diverse and complex structures. J.Solid. State. Chem. 270, 346–359. 10.1016/j.jssc.2018.11.029

[B37] Ovchinnikov A. Bobev S. (2020). Bismuth as a reactive solvent in the synthesis of multicomponent transition-metal-bearing bismuthides. Inorg. Chem. 10.1021/acs.inorgchem.9b02881. [Epub ahead of print]. 31860285

[B38] Ovchinnikov A. Darone G. Saparov B. Bobev S. (2018a). Exploratory work in the quaternary system of Ca–Eu–Cd–Sb: synthesis, crystal, and electronic structures of new zintl solid solutions. Materials 11:2146 10.3390/ma11112146 PMC6265713 30384471

[B39] Ovchinnikov A. Makongo J. P. A. Bobev S. (2018b). Yet again, new compounds found in systems with known binary phase diagrams. Synthesis, crystal and electronic structure of Nd_3_Bi_7_ and Sm_3_Bi_7_. Chem. Commun. 54, 7089–7092. 10.1039/C8CC02563K 29881846

[B40] Ovchinnikov A. Prakash J. Bobev S. (2017a). Crystal chemistry and magnetic properties of the solid solutions Ca_14−x_*RE*_*x*_MnBi_11_ (*RE* = La–Nd, Sm, and Gd–Ho; *x* ≈ 0.6–0.8). Dalton. Trans. 46, 16041–16049. 10.1039/C7DT03715E 29147719

[B41] Ovchinnikov A. Saparov B. Xia S.-Q. Bobev S. (2017b). The ternary alkaline-earth metal manganese bismuthides Sr_2_MnBi_2_ and Ba_2_Mn_1−x_Bi_2_ (*x* ≈ 0.15). Inorg. Chem. 56, 12369–12378. 10.1021/acs.inorgchem.7b01851 28968067

[B42] Papoian G. Hoffmann R. (2000). Hypervalent bonding in one, two, and three dimensions: extending the Zintl-Klemm concept to nonclassical electron-rich networks. Angew. Chem. Int. Ed. 39, 2408–2448. 10.1002/1521-3773(20000717)39:14<2408::AID-ANIE2408>3.0.CO;2-U 10941096

[B43] Raju N. P. Greedan J. E. Ferguson M. J. Mar A. (1998). LaCrSb_3_: a new itinerant electron ferromagnet with a layered structure. Chem. Mater. 10, 3630–3635. 10.1021/cm9803758

[B44] SADABS (2014). Bruker AXS Inc. Madison, WI: SADABS

[B45] SAINT (2014). Bruker AXS Inc. Madison, WI: SAINT.

[B46] Savin A. Nesper R. Wengert S. Fässler T. F. (1997). ELF: the electron localization function. Angew Chem Int Ed Engl. 36, 1808–1832. 10.1002/anie.199718081

[B47] Sheldrick G. M. (2015a). *SHELXT* – Integrated space-group and crystal-structure determination. Acta. Crystallogr. A. 71, 3–8. 10.1107/S2053273314026370 25537383 PMC4283466

[B48] Sheldrick G. M. (2015b). Crystal structure refinement with *SHELXL*. Acta. Crystallogr. C. 71, 3–8. 10.1107/S2053229614024218 25567568 PMC4294323

[B49] Steinberg S. Dronskowski R. (2018). The Crystal Orbital Hamilton Population (COHP) method as a tool to visualize and analyze chemical bonding in intermetallic compounds. Crystals 8:225 10.3390/cryst8050225

[B50] Villars P. Calvert L. D. (1991). Pearson's Handbook of Crystallographic Data for Intermetallic Phases, 2nd Edn. Materials Park, OH: American Society for Metals.

[B51] von Barth U. Hedin L. (1972). A local exchange-correlation potential for the spin polarized case: I. J. Phys. C. Solid. State. Phys. 5, 1629–1642. 10.1088/0022-3719/5/13/012

[B52] Wakiya K. Sugiyama Y. Komagata T. Uehara M. Sato H. Gouchi J. (2019). Intermediate valence state of Ce in the novel quaternary compound CeRu_2_Sn_2_Zn_18_. J. Alloys. Compd. 797, 309–313. 10.1016/j.jallcom.2019.04.345

[B53] Wang J. Yang M. Pan M.-Y. Xia S.-Q. Tao X.-T. He H.. (2011). Synthesis, crystal and electronic structures, and properties of the new pnictide semiconductors *A*_2_Cd*Pn*_2_ (*A* = Ca, Sr, Ba, Eu; *Pn* = P, As). Inorg. Chem. 50, 8020–8027. 10.1021/ic200286t 21786747

[B54] Wolff G. A. Mlavsky A. I. (1974). Travelling solvent techniques, in Crystal Growth: Theory and Techniques, Vol. 1, ed Goodman C. H. L. (Boston, MA: Springer US), 193–232.

[B55] Zaikina J. V. Griffin V. S. Latturner S. E. (2017). Switching on a spin glass: flux growth, structure, and magnetism of La_11_Mn_13−x–y_Ni_*x*_Al_*y*_Sn_4−δ_ intermetallics. Inorg. Chem. 56, 15194–15202. 10.1021/acs.inorgchem.7b02555 29182325

[B56] Zaikina J. V. Jo Y.-J. Latturner S. E. (2010). Ruthenium intermetallics grown from La–Ni Flux: synthesis, structure, and physical properties. Inorg. Chem. 49, 2773–2781. 10.1021/ic902151d 20141115

