# Supplementary material for: Exploration of Multi-Component Vanadium and Titanium Pnictides Using Flux Growth and Conventional High-Temperature Methods
Source: Front Chem. 2020 Jan 10;7:909. doi: 10.3389/fchem.2019.00909 (PMC6965498; doi:10.3389/fchem.2019.00909)
Supplement: Supplementary file 1 [file Data_Sheet_1.pdf]

Exploration of multi-component vanadium and titanium pnictides using flux growth and conventional high-temperature methods

Supporting Information

Alexander Ovchinnikov[1,2] and Svilen Bobev[1*]

[1]Department of Chemistry and Biochemistry, University of Delaware, Newark, Delaware 19716, United States

[2]Department of Materials and Environmental Chemistry, Stockholm University, Svante Arrhenius väg 16C, 10691 Stockholm, Sweden

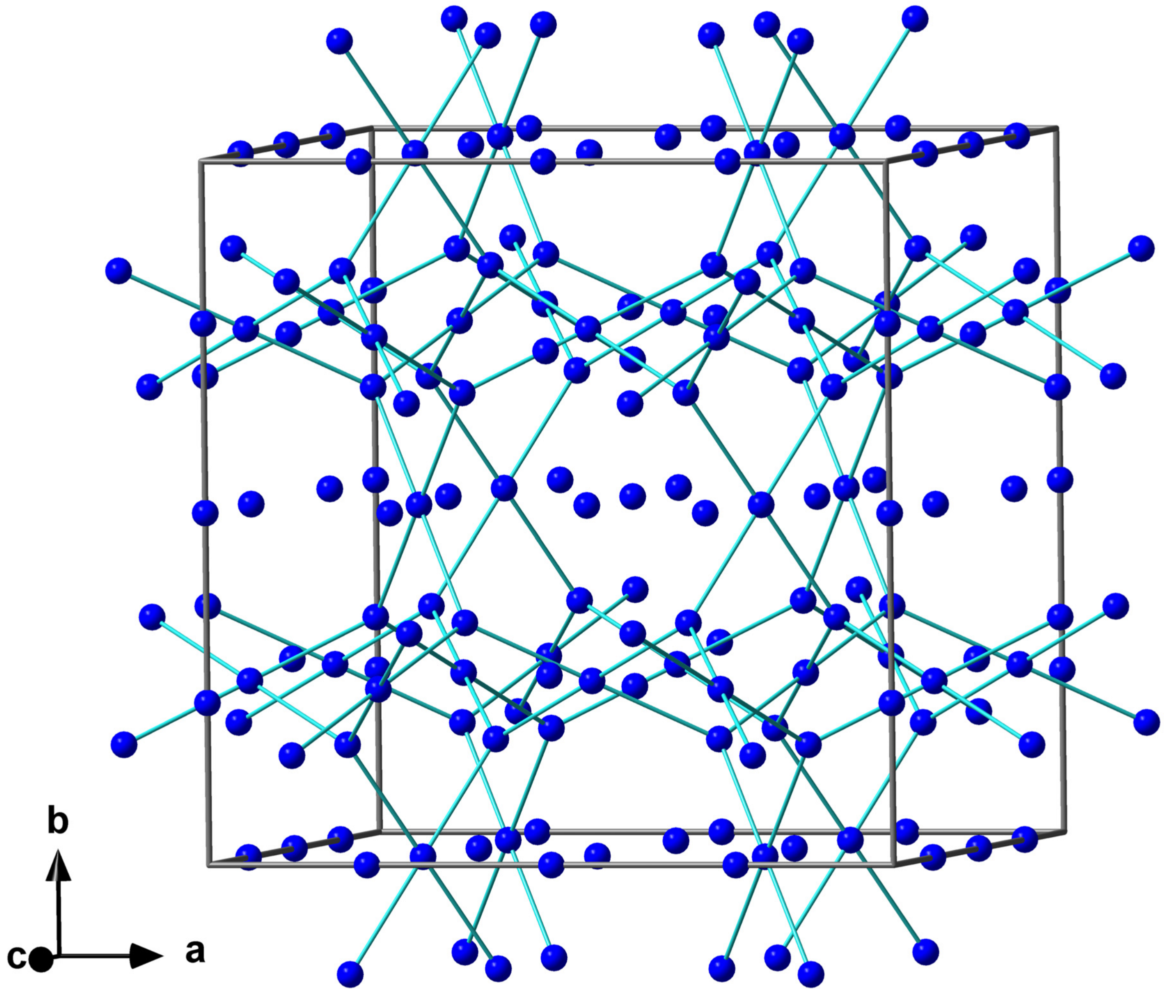

Figure S1. As substructure in $Ba_8Ti_{13-x}M_xAs_{21}$ (M = Nb, Ta). As–As contacts with $d_{As–As}$ = 3.203(2) Å (Nb) and 3.2043(8) Å are shown in cyan.

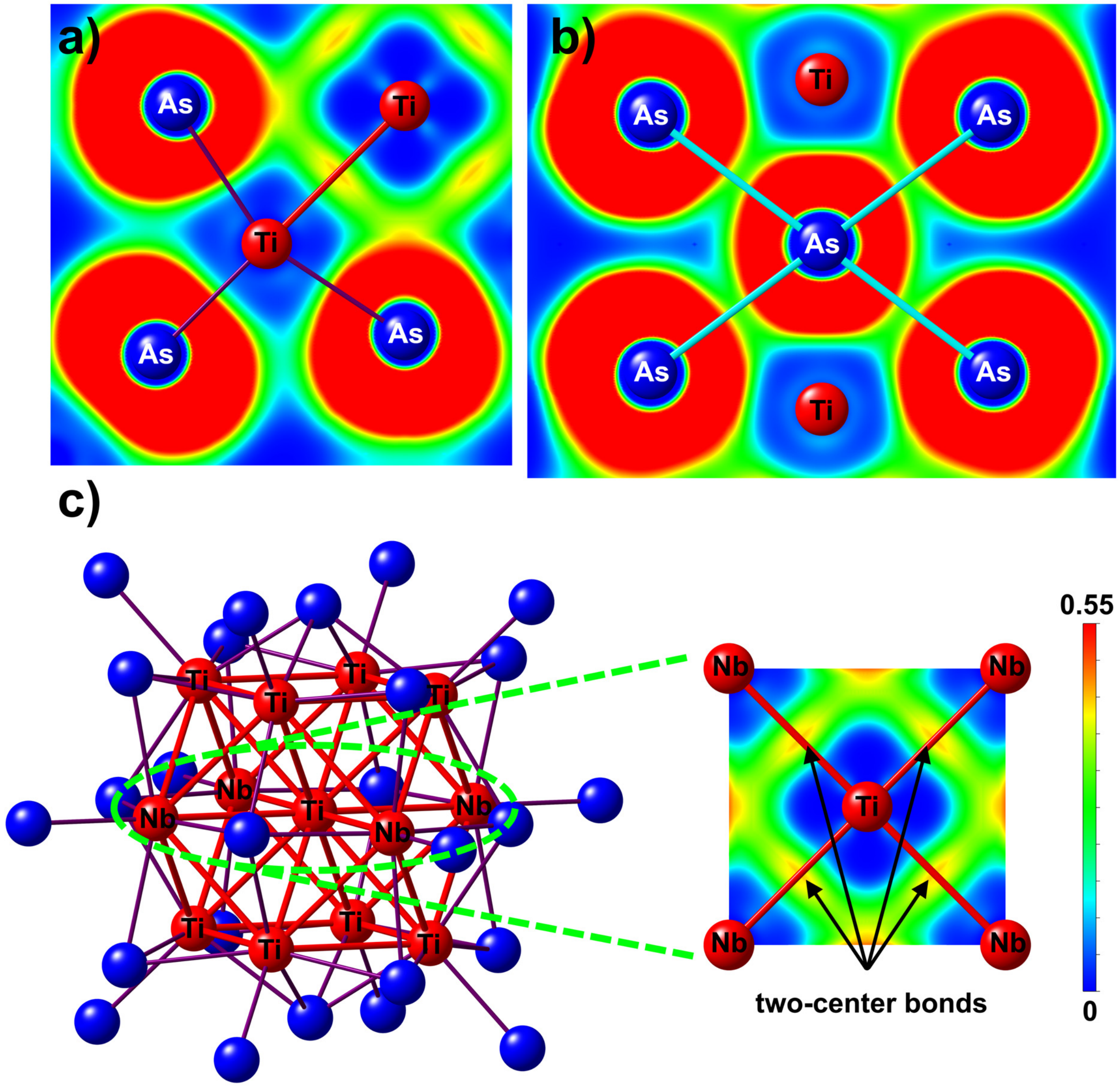

Figure S2. Selected sections of the Electron Localization Function (ELF) through Ti–As (**a**) and As–As contacts (**b**). Cuboctahedral transition metal cluster in the ordered model $Ba_8Ti_9Nb_4As_{21}$ (**c**, left) and a selected ELF section through the metal atoms (**c**, right).
